# Supplementary material for: Shrub encroachment alters microbial community composition and soil carbon and nitrogen cycling functional genes in northern peatlands
Source: Microbiol Spectr. 2025 Aug 27;13(10):e00542-25. doi: 10.1128/spectrum.00542-25 (PMC12502596; doi:10.1128/spectrum.00542-25)

Table S1 Dominant species in the herbaceous and shrub layers of peatlands at different stages of shrub invasion.

| Shrub invasion phase | Herbaceous layer dominant species (relative importance value %) | Shrub layer dominant species (relative importance value %) |
| --- | --- | --- |
|  | *Carex schmidtii* (21.25) | / |
| non-shrub invasion | *Thelypteris palustris* (15.80) | / |
|  | *Viola amurica* (11.94) | / |
|  | *Thelypteris palustris* (24.46) | *Spiraea salicifolia* (31.04) |
| shrub invasion | *Carex schmidtii* (18.21) | *Salix myrtilloides* (11.15) |
|  | *Phragmites australis*（8.01) | *Betula ovalifolia* (10.20) |
|  | *Thelypteris palustris* (20.21) | *Spiraea salicifolia* (52.89) |
| shrub invasion expansion | *Carex schmidtii* (17.23) | *Betula ovalifolia* (18.46) |
|  | *Phragmites australis* (8.01) | *Salix rosmarinifolia* (6.88) |

Table S2 Summary of raw metagenomic sequencing data

| Samples | Insert Size (bp) | Read length (bp) | Raw reads | Raw base (bp) |  |
| --- | --- | --- | --- | --- | --- |
| A13-1 | 500 | 150 | 87790776 | 13256407176 |  |
| A13-2 | 500 | 150 | 85610708 | 12927216908 |  |
| A14-1 | 500 | 150 | 84812386 | 12806670286 |  |
| A14-2 | 500 | 150 | 93828632 | 14168123432 |  |
| A17-1 | 500 | 150 | 92093436 | 13906108836 |  |
| A17-2 | 500 | 150 | 88443448 | 13354960648 |  |
| A18-1 | 500 | 150 | 92883496 | 14025407896 |  |
| A18-2 | 500 | 150 | 89976696 | 13586481096 |  |
| A20-1 | 500 | 150 | 87924752 | 13276637552 | |
| A20-2 | 500 | 150 | 85960212 | 12979992012 | |
| B4-1 | 500 | 150 | 86360068 | 13040370268 | |
| B4-2 | 500 | 150 | 90721852 | 13698999652 | |
| B16-1 | 500 | 150 | 89513416 | 13516525816 | |
| B6-2 | 500 | 150 | 88210830 | 13319835330 | |
| B7-1 | 500 | 150 | 98118256 | 14815856656 | |
| B7-2 | 500 | 150 | 88689418 | 13392102118 | |
| B8-1 | 500 | 150 | 89183452 | 13466701252 | |
| B8-2 | 500 | 150 | 87123350 | 13155625850 | |
| B11-1 | 500 | 150 | 88340160 | 13339364160 | |
| B11-2 | 500 | 150 | 87454220 | 13205587220 | |
| C1-1 | 500 | 150 | 83306906 | 12579342806 | |
| C1-2 | 500 | 150 | 87422240 | 13200758240 | |
| C5-1 | 500 | 150 | 84925262 | 12823714562 | |
| C5-2 | 500 | 150 | 88989860 | 13437468860 | |
| C10-1 | 500 | 150 | 95646852 | 14442674652 | |
| C10-2 | 500 | 150 | 88178882 | 13315011182 | |
| C12-1 | 500 | 150 | 81832918 | 12356770618 | |
| C12-2 | 500 | 150 | 83849436 | 12661264836 | |
| C18-1 | 500 | 150 | 86820282 | 13109862582 | |
| C18-2 | 500 | 150 | 81905816 | 12367778216 | |

**Note:** In the table, **A** represents non-encroached shrub plots, **B** represents shrub-encroached plots, and **C** represents shrub expansion plots. The number **1** indicates surface soil (0-30 cm), and **2** indicates subsurface soil (30-60 cm).

Table S3 Summary of sequencing data after quality control

| Samples | | Clean reads | Clean base(bp) | Percent in raw reads(%) | Percent in raw bases(%) |
| --- | --- | --- | --- | --- | --- |
| A13-1 | | 85975812 | 12879739304 | 97.93262563 | 97.1585976 |
| A13-2 | | 84026598 | 12590524196 | 98.14963567 | 97.3954741 |
| A14-1 | | 83225572 | 12464301303 | 98.12903035 | 97.32663545 |
| A14-2 | | 92171744 | 13813232397 | 98.2341339 | 97.49514439 |
| A17-1 | | 90421986 | 13547986458 | 98.1850498 | 97.42471181 |
| A17-2 | | 86841294 | 13018712336 | 98.18849894 | 97.48222162 |
| A18-1 | | 91074674 | 13661364716 | 98.05259053 | 97.40440219 |
| A18-2 | | 88353728 | 13251769739 | 98.19623517 | 97.53643821 |
| A20-1 | | 86165420 | 12902437095 | 97.9990481 | 97.18151184 |
| A20-2 | | 84504144 | 12668315478 | 98.306114 | 97.59879256 |
| B4-1 | | 84560874 | 12651897987 | 97.91663666 | 97.02100268 |
| B4-2 | | 89058716 | 13364177708 | 98.16677464 | 97.55586574 |
| B16-1 | | 87815364 | 13163101127 | 98.10301955 | 97.38524016 |
| B6-2 | | 86548498 | 12973406158 | 98.11550124 | 97.39914824 |
| B7-1 | | 96023064 | 14401965139 | 97.86462572 | 97.20642872 |
| B7-2 | | 87024366 | 13044831844 | 98.12260353 | 97.40690243 |
| B8-1 | 87251894 | | 13062342385 | 97.83417444 | 96.99734286 |
| B8-2 | 85509572 | | 12815549832 | 98.14770897 | 97.41497651 |
| B11-1 | 86389388 | | 12932909928 | 97.79174953 | 96.95297147 |
| B11-2 | 85842706 | | 12859251264 | 98.15730562 | 97.37735286 |
| C1-1 | 81544824 | | 12204476751 | 97.88483082 | 97.01998697 |
| C1-2 | 85762924 | | 12862782830 | 98.10195209 | 97.4397273 |
| C5-1 | 83380320 | | 12494321904 | 98.18082163 | 97.43137874 |
| C5-2 | 87408104 | | 13112495864 | 98.22254356 | 97.5815907 |
| C10-1 | 93616910 | | 14031130692 | 97.87766983 | 97.15050038 |
| C10-2 | 86470704 | | 12953446382 | 98.06282643 | 97.28453251 |
| C12-1 | 80180584 | | 12006491503 | 97.98084433 | 97.16528593 |
| C12-2 | 82332960 | | 12339634192 | 98.1914297 | 97.45972738 |
| C18-1 | 85200286 | | 12763073694 | 98.13408116 | 97.35474811 |
| C18-2 | 80407734 | | 12049764069 | 98.17096993 | 97.42868815 |

**Note:** In the table, **A** represents non-encroached shrub plots, **B** represents shrub-encroached plots, and **C** represents shrub expansion plots. The number **1** indicates surface soil (0-30 cm), and **2** indicates subsurface soil (30-60 cm).

Table S4 Metagenome assembly summary statistics

| Samples | Contigs | Contigs bases(bp) | N50(bp) | N90(bp) | Max(bp) | Min(bp) |
| --- | --- | --- | --- | --- | --- | --- |
| A13-1 | 962311 | 575236850 | 610 | 347 | 22385 | 300 |
| A13-2 | 1018704 | 645624759 | 649 | 354 | 73925 | 300 |
| A14-1 | 626768 | 325911252 | 518 | 337 | 39593 | 300 |
| A14-2 | 1060531 | 621258086 | 593 | 347 | 94020 | 300 |
| A17-1 | 970103 | 537052773 | 549 | 342 | 19828 | 300 |
| A17-2 | 1219517 | 773880086 | 649 | 354 | 92090 | 300 |
| A18-1 | 795875 | 443348355 | 565 | 342 | 16192 | 300 |
| A18-2 | 1221275 | 762324271 | 640 | 354 | 49710 | 300 |
| A20-1 | 716519 | 382431598 | 536 | 339 | 41083 | 300 |
| A20-2 | 1132042 | 726918025 | 656 | 354 | 202974 | 300 |
| B4-1 | 594557 | 319002199 | 540 | 340 | 10687 | 300 |
| B4-2 | 1286294 | 852076657 | 683 | 357 | 149764 | 300 |
| B6-1 | 718835 | 394914285 | 558 | 342 | 75668 | 300 |
| B6-2 | 1330596 | 871736075 | 681 | 358 | 157578 | 300 |
| B7-1 | 974404 | 563585948 | 590 | 347 | 31395 | 300 |
| B7-2 | 1181089 | 776192092 | 676 | 356 | 209381 | 300 |
| B8-1 | 858703 | 508140933 | 602 | 348 | 90132 | 300 |
| B8-2 | 1272745 | 839840837 | 685 | 358 | 100685 | 300 |
| B11-1 | 750023 | 446131657 | 602 | 348 | 35957 | 300 |
| B11-2 | 1041917 | 696798261 | 691 | 356 | 149763 | 300 |
| C1-1 | 719031 | 405951960 | 574 | 345 | 18026 | 300 |
| C1-2 | 1181692 | 760825897 | 659 | 355 | 110921 | 300 |
| C5-1 | 914912 | 520792362 | 570 | 344 | 38662 | 300 |
| C5-2 | 1237080 | 812347796 | 677 | 357 | 167697 | 300 |
| C10-1 | 777847 | 427212389 | 557 | 342 | 15031 | 300 |
| C10-2 | 1195426 | 742400830 | 640 | 353 | 116165 | 300 |
| C12-1 | 675409 | 365605190 | 546 | 342 | 19172 | 300 |
| C12-2 | 1152886 | 738666587 | 656 | 355 | 104226 | 300 |
| C18-1 | 966843 | 579977716 | 612 | 349 | 41920 | 300 |
| C18-2 | 1129549 | 708201789 | 646 | 353 | 88520 | 300 |

**Note:** In the table, **A** represents non-encroached shrub plots, **B** represents shrub-encroached plots, and **C** represents shrub expansion plots. The number **1** indicates surface soil (0-30 cm), and **2** indicates subsurface soil (30-60 cm).

Table S5 Annotated functional gene profiles from metagenomic data

| Genes | Total length (bp) | Average length (bp) | Catalog genes | Catalog total length (bp) | Catalog average length (bp) |
| --- | --- | --- | --- | --- | --- |
| 36474417 | 15429449154 | 423.02 | 10603549 | 4864589280 | 458.77 |

Table S6 Functional genes related to carbon cycling

| Genes | Total length (bp) | Average length (bp) | Catalog genes | Catalog total length (bp) | Catalog average length (bp) |
| --- | --- | --- | --- | --- | --- |
| 36474417 | 15429449154 | 423.02 | 297531 | 154574553 | 519.52 |

Table S7 Functional genes related to nitrogen cycling

| Genes | Total length (bp) | Average length (bp) | Catalog genes | Catalog total length (bp) | Catalog average length (bp) |
| --- | --- | --- | --- | --- | --- |
| 36474417 | 15429449154 | 423.02 | 31225 | 16560030 | 530.35 |

Table S8 Functional gene prediction and annotation statistics

| Samples | Predicted gene number | KEGG annotated gene number | CAZy annotated gene number |
| --- | --- | --- | --- |
| A13-1 | 3399230 | 904517 | 153577 |
| A13-2 | 3985294 | 1019338 | 194335 |
| A14-1 | 2856894 | 796137 | 127200 |
| A14-2 | 4314647 | 1118892 | 207670 |
| A17-1 | 3455428 | 929805 | 157972 |
| A17-2 | 3931115 | 1007700 | 189466 |
| A18-1 | 3535771 | 954207 | 161496 |
| A18-2 | 3939334 | 1014584 | 193469 |
| A20-1 | 3101740 | 850438 | 141341 |
| A20-2 | 3870021 | 974514 | 192777 |
| B4-1 | 2819257 | 792445 | 123895 |
| B4-2 | 3974573 | 1002473 | 194544 |
| B6-1 | 3105898 | 853668 | 141030 |
| B6-2 | 3752825 | 934301 | 190807 |
| B7-1 | 4004526 | 1072702 | 181511 |
| B7-2 | 3784324 | 966169 | 183991 |
| B8-1 | 2850205 | 783648 | 119223 |
| B8-2 | 3689228 | 925268 | 185890 |
| B11-1 | 2240567 | 640601 | 88176 |
| B11-2 | 3497645 | 902123 | 165931 |
| C1-1 | 2171614 | 609427 | 86598 |
| C1-2 | 3930357 | 995064 | 196445 |
| C5-1 | 3750574 | 990041 | 177673 |
| C5-2 | 3901292 | 981762 | 195259 |
| C10-1 | 3122217 | 875386 | 137970 |
| C10-2 | 3958440 | 1009666 | 197686 |
| C12-1 | 3497428 | 945082 | 162659 |
| C12-2 | 3749153 | 951717 | 184470 |
| C18-1 | 3603855 | 949690 | 170875 |
| C18-2 | 3821530 | 973737 | 187996 |

**Note:** In the table, **A** represents non-encroached shrub plots, **B** represents shrub-encroached plots, and **C** represents shrub expansion plots. The number **1** indicates surface soil (0-30 cm), and **2** indicates subsurface soil (30-60 cm).

Fig. S1 Length distribution of quality-controlled sequencing reads


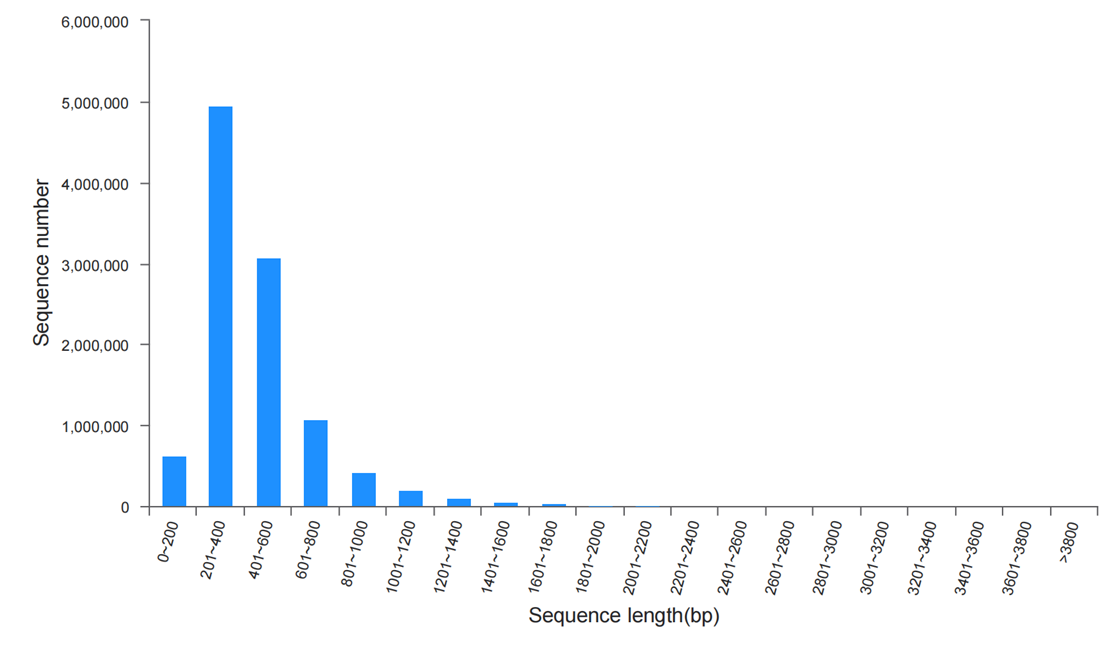

Supplement: Supplemental figure and tables — Data related to the metagenome. [file spectrum.00542-25-s0001.docx]
